# Radiation dose reduction for CT-guided intrathecal nusinersen administration in adult patients with spinal muscular atrophy

**DOI:** 10.1038/s41598-020-60240-x

**Published:** 2020-02-25

**Authors:** Isabell Cordts, Marcus Deschauer, Paul Lingor, Egon Burian, Thomas Baum, Claus Zimmer, Christian Maegerlein, Nico Sollmann

**Affiliations:** 1 Department of Neurology, Klinikum rechts der Isar, Technische Universität München, Munich, Germany; 2 Department of Diagnostic and Interventional Neuroradiology, Klinikum rechts der Isar, Technische Universität München, Munich, Germany; 3 TUM-Neuroimaging Center, Klinikum rechts der Isar, Technische Universität München, Munich, Germany

**Keywords:** Motor neuron, Computed tomography

## Abstract

Intrathecal administration of nusinersen in adult spinal muscular atrophy (SMA) patients with scoliosis and spondylodesis requires image guidance, which is preferably achieved with multi-detector computed tomography (MDCT). As long-term treatment is necessary and patients are young, radiation doses should be reduced to a minimum whilst a sufficient image quality for precise interventional performance should be kept. We compared 44 MDCT standard-dose scans (133.0–200.0 mA) with a hybrid iterative reconstruction (iDose4) to 20 low-dose scans (20.0–67.0 mA) with iterative model reconstruction (IMR), which were performed for procedure planning of intrathecal nusinersen administration in 13 adult patients with SMA and complex spinal conditions. Qualitative image evaluation, including confidence for intervention planning, was performed by two neuroradiologists for standard- and low-dose scans. All 64 MDCT-guided intrathecal administrations of nusinersen were successful. The dose length product (DLP) was significantly lower when using low-dose scanning with IMR (median DLP of standard-dose scans: 92.0 mGy•cm vs. low-dose scans: 34.5 mGy•cm; p < 0.0001). Image quality was significantly reduced for low-dose compared to standard-dose scanning. However, bone/soft tissue contrast and confidence for intervention planning were not significantly impaired in low-dose MDCT according to both readers, showing good inter-reader agreement. Thus, we hereby demonstrate a low-dose MDCT protocol combined with advanced image reconstruction for scanning during procedure planning as a viable option for image guidance in intrathecal nusinersen treatment of adult SMA patients with complex spinal conditions.

## Introduction

Spinal muscular atrophy (SMA) is an autosomal-recessive neurodegenerative disease caused by mutations in the survival motor neuron 1 (SMN1) gene^1^. The lack of SMN protein results in progressive degeneration of motor neurons causing muscle weakness and atrophy with varying severity. In June 2017, the European Medicines Agency approved nusinersen as the first drug to treat SMA. Nusinersen, an antisense oligonucleotide (ASO), does not efficiently cross the blood-brain barrier and thus has to be administered directly into the cerebrospinal fluid (CSF), which is commonly achieved by a lumbar puncture (LP).

Intrathecal drug administration presents challenges in adult SMA patients due to severe neuromuscular scoliosis and frequent preceding spinal fusion surgeries. Recent studies showed intrathecal treatment with nusinersen to be feasible and safe when using image guidance in patients with scoliosis^2,3^. Generally, guidance by computed tomography (CT) was evaluated to be superior to fluoroscopy-assistance in patients with a complex spinal architecture^2,3^. However, CT-guided interventions are accompanied by radiation exposure and, therefore, radiation risks need to be taken into account, such as an increased risk of cancer induction^4,5^. Particularly in patients with SMA efforts must be made to keep radiation exposure as low as possible since patients are young and we currently have to assume long-term therapy before other dosage forms or competing drugs become available in the future.

Of note, median radiation dose indicated as the dose length product (DLP) in SMA patients with CT-assisted procedures was previously reported to be considerably high (120.1 mGy•cm), with this value being even increased in patients with spinal fusion (246.5 mGy•cm)^6^. Our first analysis of 53 CT-guided LPs with standard-dose imaging protocols performed in 11 SMA patients revealed a median DLP of 100 mGy•cm^7^. With respect to the “as low as reasonably achievable” (ALARA) principle, lowering of radiation doses for CT is an important topic for patient safety^8,9^.

Decreases in radiation doses for CT can be realized with various approaches including lowering of tube currents during CT image acquisition, which can be efficiently combined with advanced image reconstruction approaches to preserve sufficient image quality despite reduced delivery of energy^10–12^. Nevertheless, the tube current should only be reduced to a level at which image quality still guarantees an accurate and reliable treatment, particularly in patients with demanding anatomies or implants at the level of the spine. Against this background, the present study evaluates low-dose multi-detector computed tomography (MDCT) for procedure planning of intrathecal nusinersen administration in a consecutive series of patients with SMA and complex spinal conditions.

## Materials and Methods

### Patients and study design

This monocentric study was performed according to the Declaration of Helsinki and approved by the ethical committee of the Technical University of Munich. Written informed consent was obtained from all patients. The prerequisite for a potential treatment with nusinersen by intrathecal administration was a documented mutation of the SMN1 gene. Patients were considered not eligible for intrathecal administration if contraindications for LP were present. Treatments were performed on an outpatient basis with repetitive intrathecal nusinersen applications under image guidance by MDCT on days 1, 14, 28, and 63, followed by further applications every 4 months. LPs and subsequent drug delivery were performed by two board-certified neuroradiologists with experience in the management of patients with complex spine anatomy based on >10 years of clinical diagnostic and interventional practice.

From September 2017 to October 2019, a total of 73 consecutive CT-guided intrathecal injections of nusinersen were performed in 13 patients with SMA (8 with SMA type 2 and 5 with SMA type 3). The patient cohort comprised 7 male and 6 female patients aged 16 to 46 years (mean age ± standard deviation: 30.2 ± 9.6 years) at first LP. All patients presented with severe motor impairment and none of the patients was ambulatory. Mean functional scores at baseline were 1.9 points for Hammersmith Functional Motor Scale Expanded (HFMSE), 9.6 points for Revised Upper Limb Module (RULM), and 23.7 points for Amyotrophic Lateral Sclerosis Functional Rating Scale-Revised (ALS-FRS-R). Furthermore, all patients had a scoliosis with varying severity, 7 had a history of previous spinal instrumentation, including posterior spinal fusion and growing rods.

This study used the routine MDCT imaging data as acquired for procedure planning purposes of LP for intrathecal nusinersen administration. An update of imaging protocols for CT-guided interventional procedures took place at our institution in May 2019, which included systematic lowering of tube currents combined with routine introduction of advanced image reconstruction algorithms. Out of the imaging datasets of the 73 CT-guided intrathecal injections, 64 datasets were considered in this study. The nine remaining datasets were performed with a scanner from a different vendor (due to temporary limited availability of the standardly used MDCT scanners), thus hampering direct comparability.

### Multi-detector computed tomography

#### Scanning procedure and intrathecal drug delivery

Scanning was performed in lateral, right- or left-sided position with MDCT scanners (Ingenuity Core, Ingenuity, or Brilliance; Philips Healthcare, Best, The Netherlands). After initial acquisition of a frontal and lateral scout scan, the neuroradiologist placed the field of view over the area of the planned intervention (lumbar and/or sacral spine). This was followed by helical scanning of the respective area, with the acquired scans being used for procedure planning. The neuroradiologist selected the axial section allowing for optimal discrimination of the access route to the intrathecal space, followed by marking of the route on the images on the system’s console.

A local subcutaneous infiltration with mepivacaine 2% was performed to establish local, transient anesthesia. During the subsequently performed LP under sterile conditions, patients were scanned sequentially to guarantee ideal surveillance using a foot pedal and the monitoring screens inside the CT room (step-and-shoot technique). This sequential scanning was conducted stepwise until the tip of the needle was shown intrathecally, confirmed by a last scan of the final needle position and by free drainage of CSF through the needle. Depending on individual spinal anatomy, a classic posterior interlaminar access, a neuroforaminal approach, or access via a translaminar osseous canal, drilled during the first treatment session, was chosen^13^. After drainage of 5 ml CSF, patients were then administered 12 mg nusinersen (5 ml) intrathecally, followed by removal of the needle.

#### Scanning protocols and image reconstruction

Before the end of May 2019, scanning was performed with standard-dose imaging protocols (120–140 kV and 133–200 mA) on one of the aforementioned MDCT scanners. In the context of an update of CT imaging protocols for interventional procedures at our institution, a low-dose imaging protocol was established for planning purposes of CT-guided intrathecal nusinersen administration (120 kV and 20–67 mA), with all low-dose scans being performed with an Ingenuity scanner (Philips Healthcare, Best, The Netherlands). This study only used the scans by MDCT performed for the purpose of procedure planning, the survey scans and sequential scans during intervention were not considered.

**Figure 1:**
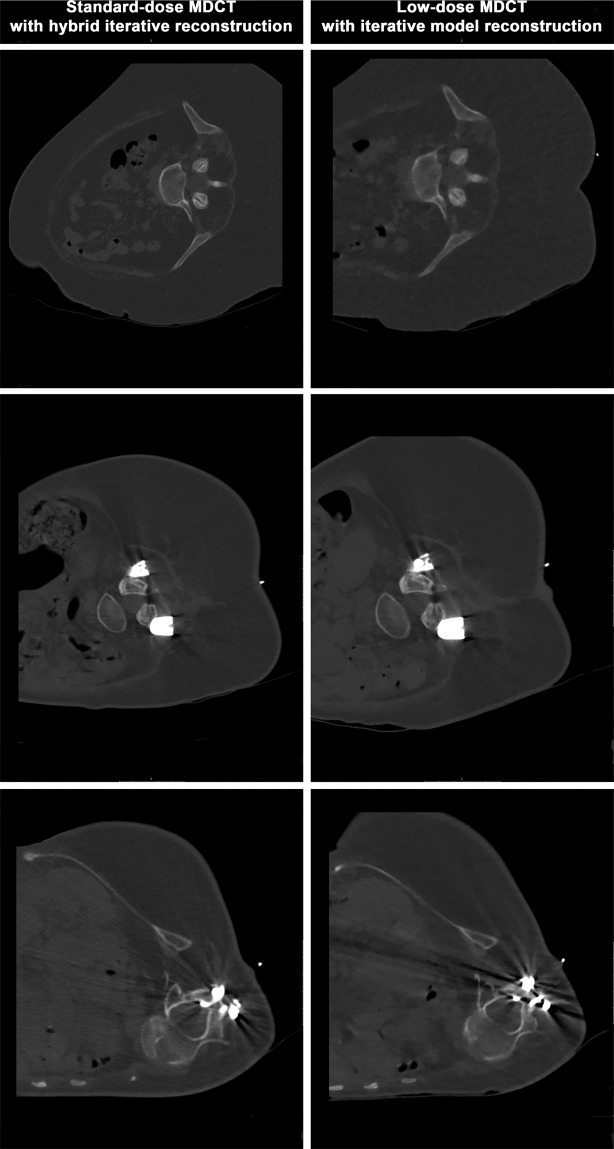
Standard- and low-dose scans for procedure planning of intrathecal nusinersen administration. This figure shows three exemplary patient cases (one patient case per horizontal column, two axial slices per patient case) using both standard-dose and low-dose scanning by multi-detector computed tomography (MDCT). Image reconstructions for standard-dose scans were performed using a hybrid iterative reconstruction algorithm (iDose4; Philips Healthcare, Best, The Netherlands). Low-dose scans were reconstructed with iterative model reconstruction (IMR; Philips Healthcare, Best, The Netherlands).

Image reconstructions for standard-dose scans were performed using the vendor’s standard hybrid iterative reconstruction algorithm (iDose4; Philips Healthcare, Best, The Netherlands; Fig. 1). In contrast, low-dose scans were all reconstructed with iterative model reconstruction (IMR; Philips Healthcare, Best, The Netherlands; Fig. 1), a model-based iterative algorithm that realizes stronger artifact and noise reductions when compared to the hybrid approach^12^. All MDCT imaging data (initial scout, helical scans for procedure planning, and sequential scanning for procedure surveillance) were sent to our hospital’s institutional digital picture archiving and communication system (PACS) after the procedures with completed image reconstructions (IDS7; Sectra AB, Linköping, Sweden).

#### Scanning and exposure reports

Reports automatically generated by the MDCT systems were reviewed to assess scanning details for standard- and low-dose scans. This included extraction of the time needed for the CT-guided LP, volumetric CT dose index (CTDIvol; in mGy), DLP (in mGy•cm), and exposure (in mAs) as well as tube voltage (in kV) and tube current (in mA) for the scans of procedure planning, and total DLP (in mGy•cm) per session summarizing the estimations for scout, helical, and sequential scanning.

### Qualitative image evaluations

The 64 MDCT image datasets acquired for procedure planning were evaluated in the PACS viewer by two independent neuroradiologists, who have not been involved in the execution of the interventional procedures (2 and 6 years of experience in neuroradiological imaging, reader 1 [R1] and reader 2 [R2]). The readers were blinded to the dose reports and clinical characteristics of the patients as well as to the readings of each other. They evaluated both the standard- and low-dose MDCT scans in a randomized order with the patient names and scanning details being hidden in the image layouts during evaluation, thus being unaware of the standard- or low-dose nature of the respective image datasets. The readers assessed overall image quality, overall artifacts, bone/soft tissue contrast, and confidence for intervention planning based on 3-point or 5-point Likert scales^14^. The scheme for qualitative image evaluations is presented in Table 1.

**Table 1:** Scoring scheme for image evaluation.

	Score						
	1	2		3	4		5
**Overall image quality**	Very good to perfect	Good to very good		Medium	Adequate		Poor
**Overall artifacts**	None	Minimal		Prominent	Major		Severe
**Bone/soft tissue contrast**	Very good to perfect	Good to very good		Medium	Adequate		Poor
	**Score**						
	**1**		**2**			**3**	
**Confidence for intervention planning**	High		Medium			Low	

### Statistical analyses

For statistical analyses, GraphPad Prism (version 6.0; GraphPad Software Inc., La Jolla, CA, USA) and SPSS (version 20.0; IBM SPSS Statistics for Windows, IBM Corp., Armonk, NY, USA) were used. First, descriptive statistics including mean and standard deviation (SD), median, and ranges were calculated for patient characteristics, the scores of qualitative image evaluation, and scanning details and measures of exposure.

To compare qualitative image evaluations in terms of overall image quality, overall artifacts, bone/soft tissue contrast, and confidence for intervention planning between standard-dose and low-dose MDCT scans for scorings of each reader, Mann-Whitney U tests were performed. Furthermore, to assess inter-reader agreement, quadratic Cohen’s kappa (κ) was calculated, which was performed between the scorings of both readers for standard-dose and low-dose MDCT scans, respectively. Time and attempts needed for the interventions, scanning details, and measures of exposure were compared between standard-dose and low-dose MDCT scans using Mann-Whitney U tests. A p-value <0.05 was considered statistically significant for all statistical testing.

## Results

Out of the 64 CT-guided injections considered in this study, 44 interventions were performed before the update of the CT imaging protocols, thus using standard-dose imaging protocols with iDose4 for reconstruction (Fig. 1). The remaining 20 injections were performed under the novel low-dose imaging protocol combined with IMR (Fig. 1).

Each patient has undergone at least one CT-guided LP using low-dose MDCT. All 64 CT-guided injections were successful regarding proper LP and intrathecal nusinersen administration. Comparing standard- and low-dose MDCT, there was no statistically significant difference regarding the number of attempts needed to reach the intrathecal space (standard-dose MDCT scans: median: 1.0 attempts, low-dose MDCT scans: median: 1.0 attempts; p = 0.5831). The average time needed for successful CT-guided LP, measured from acquisition of the scout scan to drainage of CSF, was not statistically different for standard-dose compared to low-dose MDCT (standard-dose MDCT scans: mean ± SD: 10.2 ± 5.6 min, low-dose MDCT scans: mean ± SD: 10.7 ± 5.9 min; p = 0.5392). Further clinical data including access to the intrathecal compartment and complications have been described before for a subgroup of the present cohort^13^.

### Scanning details and exposure

A comparison between scanning adjustments and measures of exposure between standard-dose and low-dose MDCT scans is shown in Table 2. Overall, values for tube current, exposure, and CTDIvol were significantly lower for the scans of procedure planning using low-dose scanning compared to standard-dose MDCT scans (p < 0.0001 each). Notably, the DLP for procedure planning was significantly lower in low-dose than in standard-dose scanning (standard-dose MDCT scans: median: 58.0 mGy•cm, low-dose MDCT scans: median: 10.0 mGy•cm; p < 0.0001) as well as for the estimations for the whole session comprising scout, helical, and sequential scanning (standard-dose MDCT scans: median: 92.0 mGy•cm, low-dose MDCT scans: median: 34.5 mGy•cm; p < 0.0001).

**Table 2:** Details and exposure for procedure planning scans.

	Standard-dose MDCT	Low-dose MDCT	p-value
Tube voltage (kV)	120.0 (120.0–140.0)	120.0 (120.0)	n.s.
Tube current (mA)	133.0 (133.0–200.0)	40.0 (20.0–67.0)	<0.0001
Exposure (mAs)	100.0 (100.0–150.0)	30.0 (15.0–30.0)	<0.0001
CTDIvol (mGy)	6.5 (6.5–13.1)	2.0 (1.0–2.0)	<0.0001
DLP (mGy•cm)	58.0 (17.0–144.5)	10.0 (4.0–24.0)	<0.0001

This table provides median and ranges for tube voltage, tube current, exposure, CTDIvol, and DLP for scans performed for procedure planning of intrathecal nusinersen administration guided by MDCT. Values derived from standard-dose scans were compared to low-dose scans using Mann-Whitney U tests, p-values indicate statistical significance (level of statistical significance: p < 0.05).

Abbreviations: MDCT, multi-detector computed tomography; n.s., not significant, CTDIvol, volumetric computed tomography dose index; DLP, dose length product.

### Qualitative image evaluations

Results for qualitative image evaluations according to scoring by both readers are provided in Table 3. Overall image quality was significantly reduced for low-dose scanning when compared to standard-dose scanning (p < 0.0001). However, scores for overall artifacts, bone/soft tissue contrast, and confidence for intervention planning were not significantly different between standard-dose and low-dose MDCT scans (p > 0.05).

**Table 3:** Results of qualitative image evaluations performed by two independent readers.

	R1			R2		
	Standard-dose MDCT	Low-dose MDCT	p-value	Standard-dose MDCT	Low-dose MDCT	p-value
Overall image quality	1.4 ± 0.5 (1–2)	2.0 ± 0.4 (1–3)	<0.0001	1.3 ± 0.5 (1–2)	1.9 ± 0.3 (1–2)	<0.0001
Overall artifacts	1.8 ± 0.7 (1–3)	2.0 ± 0.8 (1–3)	n.s.	1.7 ± 0.5 (1–3)	2.0 ± 0.8 (1–3)	n.s.
Bone/soft tissue contrast	1.1 ± 0.2 (1–2)	1.2 ± 0.4 (1–2)	n.s.	1.0 ± 0.2 (1–2)	1.1 ± 0.3 (1–2)	n.s.
Confidence for intervention planning	1.1 ± 0.2 (1–2)	1.2 ± 0.4 (1–2)	n.s.	1.1 ± 0.2 (1–2)	1.2 ± 0.4 (1–2)	n.s.

This table provides mean ± SD and ranges for the ratings of qualitative image evaluation for procedure planning of intrathecal nusinersen administration guided by MDCT. Values derived from standard-dose scans were compared to low-dose scans using Mann-Whitney U tests according to the scorings of both readers, p-values indicate statistical significance (level of statistical significance: p < 0.05).

Abbreviations: R1, reader 1; R2, reader 2; MDCT, multi-detector computed tomography; n.s., not significant; SD, standard deviation.

Inter-reader agreement was substantial for overall image quality (standard-dose MDCT scans: κ = 0.72, low-dose MDCT scans: κ = 0.79), substantial to very good for overall artifacts (standard-dose MDCT scans: κ = 0.77, low-dose MDCT scans: κ = 0.83), substantial for bone/soft tissue contrast (standard-dose MDCT scans: κ = 0.66, low-dose MDCT scans: κ = 0.77), and substantial to very good for confidence for intervention planning (standard-dose MDCT scans: κ = 0.99, low-dose MDCT scans: κ = 0.70).

## Discussion

Image guidance is a fundamental component for intrathecal nusinersen treatment in adult SMA patients with complex spinal conditions. In the present study, we introduce a low-dose imaging protocol combined with advanced image reconstruction for MDCT scanning during procedure planning of LP for intrathecal drug administration. Our approach entails a significant reduction of radiation exposure, with the median DLP showing a decrease from 58.0 mGy•cm down to 10.0 mGy•cm for the low-dose imaging protocol.

The technical procedure of CT-guided LP for intrathecal nusinersen administration is well described in the literature; however, details on the radiation exposure are often excluded^6^. Recent studies showed that radiation exposure during nusinersen administration in SMA patients is generally not higher compared to those for patients with normal spine anatomy^6,15^. Interestingly, a decrease of the radiation dose during therapy with nusinersen has been demonstrated, probably due to decreased frequency of imaging, data from successful previous injections, and a learning effect of the interventionalists^3,7,15^. Direct comparisons of radiation dosage or patient exposure between the studies is limited because different units (mSv vs. mGy•cm^15^) and different statistical values (median vs. mean^2^) were used. However, the median DLP of 100 to 120 mGy•cm given in previous studies – with values being even increased in patients with spinal fusion^6,7^ – is considerably higher compared to the median DLP of 34.5 mGy•cm for the whole session according to our study. With regard to the patients’ young age and the long-term therapy, the radiation exposure going along with CT guidance has to be addressed with respect to the ALARA principle^8,9^. Most importantly, the additional cancer risk should be taken into account and has to be discussed with the patients^4,5,16^. The cancer risk for a single injection of nusinersen with CT guidance was estimated to be rather small^6^; however, it must be stressed that the lifetime risk of cancer due to cumulative radiation exposure can stochastically increase with the dose the patient has been exposed to. Furthermore, ionizing radiation might impact gonadal function and fertility^17,18^.

Fluoroscopy is described as an alternative to CT-guided LP in SMA patients^3,15,19^, with the technique being generally associated with lower radiation exposure. Particularly for SMA patients without preceding spondylodesis but with a scoliosis impeding LP without image guidance, fluoroscopy guidance represents a viable and safe option. Moreover, in patients with a moderate spine anatomy, a switch from initial CT to fluoroscopy guidance during the course of treatment is conceivable. The use of CT imaging during the first intervention creates favorable conditions for a safe and quick first treatment that can be combined with full diagnostic imaging of the spine, with the generated CT images and data about positioning and angle of the needle providing advantageous information for later, potentially fluoroscopy-guided LPs. However, in SMA patients with spondylodesis and complex scoliosis, CT guidance seems to remain the best option for guaranteeing a safe intrathecal treatment as CT imaging provides better visualization of bony structures, muscles, and nerves with better contrast in comparison to fluoroscopy, thus allowing a very precise and safe needle targeting. Regarding MDCT as applied in the present study, image quality was lower when using the novel low-dose imaging protocol compared to standard-dose MDCT; however, scores for overall artifacts, bone/soft tissue contrast, and confidence for intervention planning were not significantly different between protocols. Moreover, CT-guided drug administration performed with low-dose imaging for planning was successful in all patients and the duration of LP was similar to the interventions with standard-dose MDCT. This indicates preserved clinical value for low-dose MDCT used for procedure planning. As spinal anatomies of our SMA patients are particularly demanding, even necessitating alternative routes like translaminar drilling, the successful intrathecal drug administration itself can be considered as evidence for a sufficient image quality and appropriate planning also when using low-dose MDCT scanning.

Image-guided LP plays a key role for many diagnostic and therapeutic interventions. Firstly, in the field of neurology, other ASOs for the treatment of genetic diseases arise, many of them requiring intrathecal administration, e.g. in Huntington’s disease or familial amyotrophic lateral sclerosis^20^. But also beyond that, a considerable shift of the LP burden to radiologists has been observed over the last two decades, with radiologists performing 46.6% of diagnostic and therapeutic LPs in a study analyzing 97,246 LPs performed in the United States^21^. Reasons for the need of image guidance are prior surgeries, extensive degenerative changes at the level of the spine, or the inability to identify osseous landmarks routinely used to plan LP, such as present in obese patients^22,23^. Moreover, LP is required for many therapeutic reasons, e.g. to instill intrathecal chemotherapy for leptomeningeal cancer treatment or prophylaxis^24^. Therefore, dose reduction is of great importance in a large range of patients undergoing various diagnostic or therapeutic interventions requiring image-guided LP.

A limitation of this study is the relatively small patient cohort consisting of 13 patients with a diagnosis of SMA, with only 20 CT-guided injections out of the 64 sessions being performed with the use of the low-dose imaging protocol for efficient procedure planning. However, after a substantial reduction of radiation exposure was realized and each patient had undergone at least one CT-guided LP using low-dose MDCT, no more patients were included for this analysis. Furthermore, SMA is a comparatively rare condition, thus restricting study cohort sizes to rather low numbers. Second, low-dose imaging protocols combined with IMR have been established for procedure planning only and not for the sequential scans acquired during the subsequent approach of CT-guided LP with intrathecal drug administration. Although similar results should be achievable for the step-and-shoot scans, future studies may include evaluations for such sequential scans during interventional procedures. Of note, realization of low-dose MDCT with advanced image reconstructions also during the interventional procedures may considerably lower radiation exposure not only for the patient but also for the personnel conducting the interventions, who are exposed to relevant cumulative doses^25,26^. Third, this study achieved lowering of radiation exposure by reductions of tube current in combination with IMR, but did not evaluate other modern approaches to limit radiation exposure, such as sparse sampling. The technique of sparse sampling has shown high potential for even more drastic dose reductions with largely preserved image quality at the spine when compared to decreases in tube current^27,28^; however, this has not yet been explicitly confirmed for MDCT scanning for planning purposes of neuroradiological interventions. While standard, commercially available MDCT scanners are not yet capable of using sparse sampling, first prototypes have already been constructed successfully^29,30^. This may pave the way for more advanced techniques that can further lower radiation exposure during clinical routine in the future.

## Conclusions

We demonstrate a low-dose imaging protocol combined with advanced image reconstruction for MDCT scanning during procedure planning of LP as a viable option for image guidance in intrathecal nusinersen treatment of adult SMA patients with complex spinal conditions. Because of young patients’ age and the long-term treatment, lowering of radiation exposure to a minimum is crucial. Despite severe anatomical hazards, the low-dose MDCT protocol showed preserved adequate image quality and planning confidence required for a successful and safe treatment of SMA patients. We encourage other centers to consider low-dose imaging protocols as an option for MDCT-guided LP in diagnostic and therapeutic management. However, fluoroscopy guidance might reflect an alternative particularly in patients with only moderate spinal complexity and without spondylodesis that should be individually considered.
